# Capecitabine and bevacizumab as first-line treatment in elderly patients with metastatic colorectal cancer

**DOI:** 10.1038/sj.bjc.6605663

**Published:** 2010-04-27

**Authors:** J Feliu, M J Safont, A Salud, F Losa, C García-Girón, C Bosch, P Escudero, R López, C Madroñal, M Bolaños, M Gil, A Llombart, J Castro-Carpeño, M González-Barón

**Affiliations:** 1 Medical Oncology Department, Hospital La Paz/Autónoma University School of Medicine. IdiPAZ. RETIC, P° de la Castellana, Madrid 261- 28046, Spain; 2 Medical Oncology Department, General Hospital, Avda.Tres Cruces, Valencia s/n - 46014, Spain; 3 Medical Oncology Department, Arnau Vilanova Hospital, Avda. Alcalde Rovira Roure, Lérida 80 – 25198, Spain; 4 Medical Oncology Department, L’Hospitalet Hospital, Av. de Josep Molins, Barcelona 29-41- 08906, Spain; 5 General Yagüe Hospital, Avda. del Cid Campeador, 96- 09005 Burgos, Spain; 6 Medical Oncology Department, Dr Peset Hospital, Avda. Gaspar Aguilar, Valencia 90- 46017, Spain; 7 Medical Oncology Department, University Lozano Blesa Hospital, Avda. San Juan Bosco, Zaragoza 15- 50009, Spain; 8 Medical Oncology Department, University Clinic Hospital, Travesía de la Choupana, Santiago de Compostela s/n - 15706, Spain; 9 Medical Oncology Department, Corochán Clinic, C/ Buigas, Barcelona 19- 08017, Spain; 10 Medical Oncology Department, San Pedro de Alcántara hospital, Avda. Millán Astray-, Cáceres 10002, Spain

**Keywords:** capecitabine, bevacizumab, colorectal neoplasms, neoplasm metastasis, aged

## Abstract

**Background:**

The efficacy and safety of capecitabine and bevacizumab in elderly patients with metastatic colorectal cancer (mCRC) considered unsuitable for receiving first-line chemotherapy with an irinotecan or oxaliplatin-based combination were assessed in a phase II, open, multicentre, uncontrolled study.

**Methods:**

Treatment consisted of capecitabine 1250 mg m^−2^ (or 950 mg m^−2^ for patients with a creatinine clearance of 30–50 ml min^−1^) twice daily on days 1–14 and bevacizumab (7.5 mg kg^−1^) on day 1 every 3 weeks.

**Results:**

A total of 59 patients aged ⩾70 years with mCRC were enrolled. In an intention-to-treat analysis, the overall response rate was 34%, with 71% of patients achieving disease control. Median progression-free survival and overall survival were 10.8 months and 18 months, respectively. In all, 32 patients (54%) had grade 3/4 adverse events (AEs), the most common being hand–foot syndrome (19%), diarrhoea (9%) and deep venous thrombosis (7%). Four patients died because of treatment-related AEs. A relationship was detected between creatinine clearance ⩽50 ml min^−1^ and the development of non-bevacizumab-related grade 3/4 AEs. The incidence of bevacizumab-associated AEs (hypertension, thromboembolic events and proteinuria) was consistent with that of previous reports in elderly patients.

**Conclusion:**

Bevacizumab combined with capecitabine represents a valid therapeutic alternative in elderly patients considered to be unsuitable for receiving polychemotherapy.

Colorectal cancer (CRC) is the third most frequent tumour in the world, with one million new cases being diagnosed every year (Parkin *et al*, 2005). Its incidence increases considerably with age, with 18.6 cases per 100 000 inhabitants per year among those <65 years of age compared with 307.3 cases per 100 000 inhabitants per year among those >65 years of age (Ries *et al*, 2002). In Europe, the median age of patients diagnosed with CRC is within the seventh decade of life and 40% of them are older than 74 years (Gatta *et al*, 1998). Furthermore, the number of elderly individuals diagnosed with CRC will probably increase in the upcoming years if the demographic forecast on population ageing is taken into account.

Treatment of advanced CRC in the elderly is still a challenge. Elderly individuals constitute a very heterogeneous population with regard to their overall health condition, functional dependence grade, co-morbidities and performance status (PS); hence, the therapeutic decisions in this population must be individualised.

Although most authors agree that the fit elderly patient should receive the same treatment as the youngest (Kohne *et al*, 2008), there is more disagreement regarding the best treatment for the unfit elderly, as conventional treatments may cause higher toxicity in them. In these cases, some therapeutic guidelines recommend treatment with monotherapy, such as capecitabine or infusions of 5-fluorouracil (5-FU) modulated with leucovorin (LV), with the possible addition of bevacizumab (National Comprehensive Cancer Network (NCCN), 2008). In fact, the subgroup analysis of a randomised phase II study comparing 5-FU–LV with 5-FU–LV plus bevacizumab suggested that the effect of bevacizumab was particularly beneficial in patients with an Eastern Cooperative Oncology Group (ECOG) PS >0, age >65 years and albumin ⩽3.5 g 100 ml (Fernando and Hurwitz, 2003), characteristics that are often observed among the elderly. In addition, in a subsequent randomised phase II study with 5-FU–LV–bevacizumab combination *vs* 5-FU–LV in patients considered unsuitable for polychemotherapy with irinotecan, a higher overall response rate (ORR; 26 *vs* 15.3%, respectively; *P*=0.055), higher median progression-free survival (PFS; 9.2 *vs* 5.5 months, respectively; *P*=0.0002) and higher overall survival (OS; 16.6 *vs* 12.9 months, respectively; *P*>0.05) were observed for the combination (Kabbinavar *et al*, 2005).

Capecitabine (Xeloda; Hoffmann-La Roche, Nutley, NJ, USA) is an oral fluoropyrimidine that has efficacy similar to that of 5-FU–LV in bolus as first-line treatment of advanced or metastatic CRC (mCRC; Hoff *et al*, 2001; Van Cutsem *et al*, 2001). Results from a previous study by our group suggested that it was also well tolerated in patients over 70 years of age with mCRC, in whom polychemotherapy was not appropriate (Feliu *et al*, 2005). Even though the addition of bevacizumab to capecitabine may be an interesting therapeutic option for these patients, the data published so far on its efficacy and safety are scarce (Puthillath *et al*, 2009).

The purpose of this phase II study was to assess the efficacy and safety of a bevacizumab–capecitabine combination in the unfit elderly patient with mCRC.

## Materials and Methods

### Patient eligibility

The main inclusion criteria were: histologically confirmed mCRC, age ⩾70 years, ECOG PS ⩽2, life expectancy of more than 3 months, ⩾1 measurable lesion according to the Response Evaluation Criteria in Solid Tumours (RECIST; Therasse *et al*, 2000) by computed tomography scan and unsuitability for receiving combination with oxaliplatin or irinotecan chemotherapy as per clinical judgment (e.g., patients with ⩾2 co-morbidities according to the Charlson co-morbidity scale (Charlson *et al*, 1987) or dependence for any of the basic or instrumental activities of daily living (ADL or IADL); Katz *et al*, 1963; Lawton, 1988). Patients who were disease free for at least 6 months after completion of adjuvant/neoadjuvant chemotherapy were eligible. Earlier radiotherapy for mCRC was permitted if completed at least 4 weeks before study inclusion and if untreated measurable disease remained. Patients were required to have adequate haematological, hepatic and renal function.

The main exclusion criteria were: operable mCRC before chemotherapy for advanced disease or earlier bevacizumab; CNS metastasis; clinically significant cardiac disease within the past 12 months; lack of physical integrity of the upper gastrointestinal tract or malabsorption syndrome; major surgical procedures or open biopsy, or having experienced significant traumatic injury within 28 days before study entry; serious non-healing wound, ulcer or bone fracture; clinical use of full-dose anti-coagulants or thrombolytics; significant bleeding diathesis or coagulopathy; and proteinuria ⩾500 mg every 24 h.

The study was conducted after approval by the appropriate independent ethics committee of each site and in accordance with the Declaration of Helsinki, Good Clinical Practices and local ethical and legal requirements. All patients provided written informed consent according to local ethics committee regulations.

### Treatment plan

The initial dose of capecitabine was determined according to the patient's renal function (creatinine clearance; CrCl). Patients received a capecitabine dose of 1250 mg m^−2^ twice daily (2500 mg m^−2^ total daily dose) if their CrCl >50 ml min^−1^ and up to 950 mg m^−2^ twice daily (1900 mg m^−2^ total daily dose) if they had a CrCl of 30–50 ml min^−1^. Capecitabine was administered for 2 weeks, followed by 1 week of rest. Bevacizumab was administered as a 30–90 min intravenous infusion at a dose of 7.5 mg kg^−1^ on day 1 of a 3-week cycle.

The Cockcroft–Gault formula (Cockcroft and Gault, 1976) was used to calculate CrCl levels between cycles. If clearance was <30 ml min^−1^, treatment was stopped. Cycles were repeated every 3 weeks for a minimum of three per patient, unless disease progression was noted. Patients with a complete response (CR), partial response or stable disease continued receiving chemotherapy until progression or detection of unacceptable adverse events (AEs).

The administration of bevacizumab was permanently discontinued in case of grade ⩾3 thromboembolic events, grade ⩾3 bleeding or uncontrolled hypertension. In case of grade ⩾3 proteinuria, treatment was withheld until proteinuria improved to <2 g every 24 h. Dose reductions for grade 2–4 AEs were carried out for capecitabine as previously described (Van Cutsem *et al*, 2001).

### Study assessments

A screening evaluation was performed at least 3 weeks before the start of treatment, including a complete anamnesis, physical examination, a routine blood analysis (haematology and biochemistry), carcinoembryogenic antigen measurement, electrocardiogram and imaging studies (chest X-ray; CT of chest, abdomen or pelvis; abdominal ultrasound or bone scan as needed), qualitative proteinuria analysis and ECOG PS. The Charlson co-morbidity scale (Charlson *et al*, 1987) and the Katz and Lawton ADL or IADL indices (Katz *et al*, 1963; Lawton, 1988) were used to assess patients’ co-morbid burden and general functional status at baseline. Before each treatment cycle, patients’ ECOG PS, vital signs, blood biochemistry and qualitative proteinuria analysis were repeated until final visit.

Tumour response was evaluated radiologically every 9 weeks (three cycles) or sooner if clinically indicated (together with carcinoembryogenic antigen measurement) during therapy, and every 12 weeks during the follow-up period. The same imaging technique was used throughout the study. RECIST v.1.0 response guidelines were used (Therasse *et al*, 2000) to define all responses after at least 9 weeks of therapy as follows: CR, partial response, stable disease or progressive disease. Disease control was defined as the sum of patients achieving a CR, partial response or stable disease. Confirmation of all responses was required after 4 weeks. Progression-free survival was defined as the time from the date of first treatment cycle to the first documentation of progressive disease or death by any cause. Overall survival was the time elapsing from the date of the first cycle of treatment until death or last known follow-up.

Patients were evaluated for AEs during therapy and until 28 days after the last study drug dose. Adverse events were graded according to the National Cancer Institute Common Toxicity Criteria (NCI CTCAE, version 3.0). For hand–foot syndrome, the previously published grading system was used (Blum *et al*, 1999).

### Statistical methods

The primary objective was to determine the ORR of the bevacizumab–capecitabine regimen in the intention-to-treat population. Secondary objectives were to analyse the PFS, OS and safety profile of the combination.

An optimal two-stage design as described by Simon, (1989) was used. Assuming a minimum efficacy of 15%, we proposed an achievement of a 30% response rate with the study combination, at a level of significance of 95% (*α* error=0.05) and a statistical power of 80% (*β* error=0.20). Assuming that 10% of patients would not be assessable, a total of 59 patients were included.

As exploratory analyses, a univariate analysis was used to compare the rate of grade 3/4 AEs according to age (70–79 years *vs* ⩾80 years), gender, CrCl (<50 *vs* ⩾50 ml min^−1^), Charlson co-morbidity scale (0 *vs* ⩾1), ECOG PS (0 *vs* ⩾1) and ADL and IADL (independent *vs* dependent). Efficacy rates (response rate, PFS and OS) according to the Charlson co-morbidity scale were also compared. Wilcoxon's signed-rank test (to compare quantitative variables) and Fisher's exact test (to compare percentages) were used. The OS and PFS values were calculated using the Kaplan–Meier method.

## Results

### Patients’ characteristics

Between August 2006 and January 2008, 59 patients, aged ⩾70 years, with recurrent or mCRC were enrolled in the study. The patients’ characteristics are shown in Table 1. A total of 22 patients (37%) had metastasis at diagnosis. Eight patients (13%) had two or more co-morbidities.

### Treatment exposure

A total of 416 treatment cycles with a mean (±s.d.) of 7.1 (±6.5) cycles per patient were administered. All of the patients enrolled in the study received at least one dose of the study medication and were considered evaluable for safety. The mean dose intensity of capecitabine was 14.6 g m^−2^ per week, this being equivalent to 94.2% of the foreseen dose intensity; it was 7.47 mg kg^−1^ per cycle for bevacizumab, which is equivalent to 99.02% of the scheduled dose intensity.

Capecitabine dose reduction/discontinuation was required in 35 (59%) patients because of AEs, the most frequent being non-haematological toxicities, principally, hand–foot syndrome and diarrhoea. Other causes included laboratory abnormal values, such as decrease in CrCl <30 mg 100 ml^−1^ and thrombocytopenia/anaemia. A total of 14 patients (24%) required at least one bevacizumab dose discontinuation because of AEs, mainly, hypertension, thromboembolism, proteinuria, haemorrhage and weight loss, all with a similar incidence rate.

Reasons for discontinuation of treatment were as follows: disease progression in 25 patients (43%), AEs in 11 (19%) patients, death for non-tumour causes in 6 patients (10%), patient refusal in 5 (9%), protocol violation in 1 (2%) and other reasons in 10 (17%).

### Efficacy

Out of the 59 patients enrolled in the study, 53 were considered to be evaluable for response. Five patients died and one patient discontinued study treatment because of AEs before completing the first 3 months of treatment and before response had been evaluated. However, they were included in the efficacy analysis as treatment failures in an intention-to-treat analysis (Table 2). Overall response rate was 34% (95% confidence interval, 22.4–47.5%), including one patient (2%) with CR and 19 patients (32%) with partial response. A further 22 patients (37%) achieved stable disease, giving a disease control rate of 71%. A total of 11 patients (19%) experienced progressive disease.

**Figure 1:**
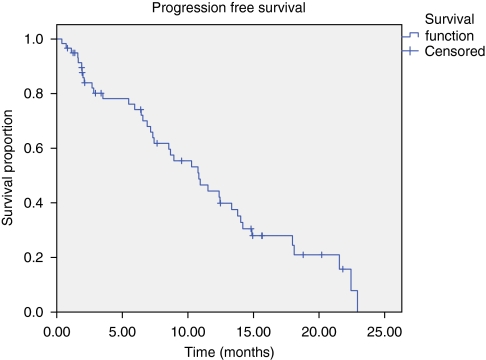
Progression-free survival.

**Figure 2:**
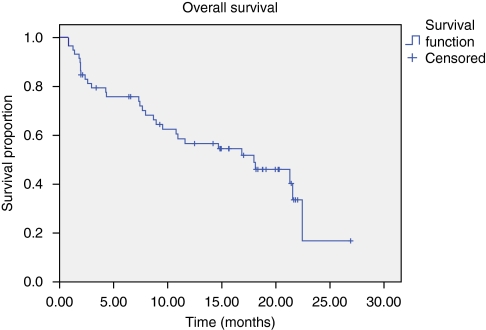
Overall survival.

Progression-free survival median was 10.8 months (95% confidence interval, 7.6–14.1 months; Figure 1), median OS was 18 months (95% confidence interval, 9.6–26.3 months; Figure 2). No correlation between response rate, PFS or OS and co-morbidity at baseline was observed.

A total of 13 patients received second-line chemotherapy after progression, which consisted of oxaliplatin- (*n*=7) or irinotecan (*n*=6) with or without cetuximab. All these patients had no co-morbidities (as per Charlson scale) along with an acceptable IADL and ADL index at baseline, and their tolerability to study treatment was good.

### Safety

Out of 59 patients, 57 (97%) reported at least one treatment-related emergent AE. The majority (74%) of treatment-related AEs were considered to be of grade 1/2. Hand–foot syndrome, diarrhoea, asthenia, pain, mucositis and arterial hypertension were the most frequent (>20%) treatment-related AEs reported (Table 3). In all, 32 patients (54%) experienced grade 3/4 AEs, the most common being hand–foot syndrome (19%) and diarrhoea (9%). A total of 12 (20%) patients experienced treatment-related arterial hypertension, this being grade 3 in one patient (2%). Furthermore, four (7%) patients had grade 3 treatment-related deep venous thrombosis and two (3%) patients had grade 1 epistaxis. No arterial thrombotic events (acute myocardial infarction, acute cerebrovascular accident or peripheral arterial thrombosis) were reported. Nine patients (15%) died within the first 60 days of the study: four (7%) were due to progressive disease and five died as a result of toxicity, which was considered treatment related in four (mucositis, digestive haemorrhage, haematologic toxicity and sepsis).

No correlation between the development of grade 3/4 AEs and age, ECOG, co-morbidity, IADL and ADL at baseline was observed. A higher frequency of bevacizumab-non-related (excluding hypertension, bleeding, proteinuria and arterial or venous thrombotic phenomena) grade 3/4 AEs was noted in those cycles in which CrCl was ⩽50 ml min^−1^ (23 *vs* 13% *P*<0.05). Furthermore, the CrCl mean value was significantly lower in cycles in which a grade 3/4 AE was reported than in cycles not reporting this AE grade (55 *vs* 62 ml min^−1^, respectively; *P*<0.05).

## Discussion

This is the first complete phase II study that analyses the efficacy and tolerability of bevacizumab combined with capecitabine chemotherapy in the elderly with mCRC. The efficacy of this regimen was noteworthy, with a 34% ORR, a disease control achievement in 71% of the patients and a median PFS and median OS of 10.8 and 18 months, respectively. These results seem to be better than those reported with the 5-FU–LV–bevacizumab regimen (ORR 26%, median PFS: 9.2 months and median OS: 16.6 months; Kabbinavar *et al*, 2005). Furthermore, they substantiate the results of a recent underpowered study by Puthillath *et al*, (2009) in 16 elderly subjects with mCRC treated with biweekly bevacizumab–capecitabine, in which a 25% ORR, a median PFS of 9.5 months and a median OS of 21 months were achieved. It should be noted that a 24% ORR and a median PFS of 7 months were achieved in a previous study conducted by our group in elderly subjects treated with capecitabine monotherapy (Feliu *et al*, 2005). Thus, bearing in mind the need for caution when results from different studies are compared, the current results support the beneficial effect of adding bevacizumab to first-line chemotherapy or capecitabine monotherapy for mCRC.

These results should be considered within the context of the elderly population studied and should be extrapolated with caution to the unfit geriatric population not included in clinical trials. In our study, a specific criterion to be eligible was the unsuitability to receive polychemotherapy because of the greater susceptibility of this patient population to treatment-related AEs. In fact, our patients often had some form of associated co-morbidity or a suboptimal IADL or ADL score. Nevertheless, there were also a number of patients not fulfilling any of these characteristics included, as they were not considered to be optimal for polychemotherapy merely on the basis of physician's subjective criteria. Furthermore, although unsuitability for receiving combination chemotherapy was an inclusion criterion, a few patients received these agents as second-line treatment according to clinician judgment in view of their good co-morbidity status at baseline and their good tolerability to the study drugs. Similar to other authors (Kabbinavar *et al*, 2005), we consider that these patients, despite their unfavourable characteristics, may benefit from less-aggressive therapeutic alternatives. In this respect, a recent phase III randomised study (Tebbutt *et al*, 2009) in relatively elderly patients with unresecable mCRC previously untreated also found that the addition of bevacizumab to capecitabine significantly improved PFS without either significant toxicity or impairment in their quality of life.

Overall, the toxicity observed with the bevacizumab–capecitabine combination in the current study was acceptable, with hand–foot syndrome and diarrhoea being the most commonly reported related AEs. Although up to 54% of the patients reported grade 3/4 AEs, this percentage was much lower than the 87% reported with the 5-FU–LV–bevacizumab regimen in a population of patients with similar characteristics (Kabbinavar *et al*, 2005). This is probably due to the lower rate of grade 3/4 diarrhoea observed in our study (9%) compared with that reported by (39%). Hand–food syndrome, which may be of special importance in this patient population as it may contribute to falls in the elderly, was reported by 46% patients in our study, and was rated grade 3/4 in 19% of patients. The frequency of these AEs was similar to that reported in another study with the same bevacizumab–capecitabine regimen in patients with advanced breast cancer (Miller *et al*, 2005). With regard to bevacizumab-associated AEs, even though 61% of patients had been previously diagnosed with hypertension, only one (3%) reported treatment-related grade 3 hypertension. No grade 4 hypertension was reported. The proportion of patients who developed grade 3 deep venous thrombosis (7%) did not differ from that reported in other studies (Kabbinavar *et al*, 2005; Miller *et al*, 2005). Although age ⩾65 years and a history of arteriosclerosis are risk factors for arterial thromboembolic events during treatment with bevacizumab (Scappaticci *et al*, 2007), this type of event was not reported in our study. Thus, age alone should not preclude patients with mCRC from receiving bevacizumab-containing therapy, and the risk–benefit balance must be weighted carefully for each patient individually. In fact, in a retrospective analysis of pooled cohorts of older patients from two studies on bevacizumab in mCRC, the risks for bevacizumab-associated events did not seem to be greater than those seen in younger patients (Kabbinavar *et al*, 2009). Moreover, the benefit derived by older patients with mCRC on adding bevacizumab to first-line chemotherapy was similar to that of younger patients without substantial increase in toxicity, according to the results of the bevacizumab Expanded Access Trial (BEAT; Van Cutsem *et al*, 2009).

A relationship was found between renal function before the administration of each chemotherapy cycle and subsequent reporting of grade 3/4 AEs. Thus, caution should be exercised in this group of vulnerable elderly subjects regarding their renal function, and CrCl should be calculated before each chemotherapy cycle. Furthermore, administration of capecitabine in elderly patients with CrCl ⩽50 ml min^−1^ should be considered on an individualised basis, as there is a greater risk of AEs (23% grade 3/4 AEs), even though the dose is reduced according to the common recommendations. In addition, clear instructions must be provided both to the patient and to his/her caregiver on the management of acute AEs, such as diarrhoea, mucositis or fever through regular telephone contact with their doctor or nurse.

Recently, results of some randomised studies in mCRC point to a similar survival rate irrespective of whether a first-line polychemotherapy is used in the first instance and treatment is changed to monotherapy after progression, or monotherapy (e.g., 5-FU or capecitabine) is used in the first instance, followed by polychemotherapy on progression (Koopman *et al*, 2007; Seymour *et al*, 2007a, 2007b). In reality, no significant differences between the different chemotherapy regimens (5-FU plus oxaliplatin, capecitabine plus oxaliplatin, 5-FU–LV monotherapy or capecitabine monotherapy) were detected in PFS or in OS in a recent study (FOCUS 2) conducted in 466 elderly or frail patients with mCRC, despite the greater response rate observed with polychemotherapy than with monotherapy (42 *vs* 39% *vs* 15 *vs* 15%, respectively; Seymour *et al*, 2007a, 2007b). Thus, when palliative treatment is the objective, it is reasonable to begin with monotherapy and then to consider a second line of polychemotherapy after progression. In that respect, the bevacizumab–capecitabine combination might be an interesting option in selected patients.

In conclusion, our results suggest that elderly patients with some vulnerability criterion (prefrail) unsuitable for receiving first-line polychemotherapy may benefit from bevacizumab in combination with capecitabine with an acceptable toxicity profile. Nevertheless, the most suitable therapeutic regimen for this group of patients is still pending and future studies particularly designed for this elderly population are needed.

## Figures and Tables

**Table 1:** Baseline patient characteristics

**Patient characteristics**	**Median (range)**	***n* (%)**
Age (years)	75 (73–79)	
		
*Range*		
70–74		27 (46)
75–79		19 (32)
⩾80		13 (22)
		
*Gender*		
Male		34 (58)
Female		25 (42)
		
*Previous adjuvant treatment*		
Chemotherapy		7 (12)
Chemotherapy and Radiotherapy		4 (7)
Radiotherapy		1 (2)
		
*ECOG PS*		
0		26 (44)
1		31 (53)
2		2 (3)
		
*Primary tumour location*		
Colon		37 (63)
Rectum		15 (25)
Rectum and Colon		7 (12)
		
*No. of metastatic sites*		
1		32 (54)
2		22 (37)
⩾3		5 (9)
		
*Localization of the metastases* a		
Liver		50 (85)
Lung		27 (46)
Others		14 (24)
		
*Compromised ADL (moderate to total dependence)*		
Lawton scale (IADL)		19 (32)
Barthel scale (ADL)		8 (14)
		
*Co-morbidities*		
Hypertension		36 (61)
Venous thrombosis		3 (5)
Cardiac disease		3 (5)
Acute cerebrovascular accident history		2 (3)
		
*Charlson co-morbidity scale*		
0		22 (37)
1		29 (50)
2		6 (10)
⩾3		2 (3)

Abbreviations: ECOG PS=Eastern Cooperative Oncology Group performance status; ADL=activities of daily living; IADL=instrumental activities of daily living.

a Each patient may have more than one location.

**Table 2:** Best response to treatment

**Response**	**No. of patients**	**%**
Complete response	1	2
Partial response	19	32
Stable disease	22	37
Progressive disease	11	19
Not evaluable^a^	6	10

a Patients included as treatment failures in the intention-to-treat analysis.

**Table 3:** Most common (⩾5%) treatment-related AEs per patient

	**Grade 1/2**	**Grade 3/4**
**NCI-CTCAE toxicity**	***n* (%)**	***n* (%)**
Hand-foot syndrome	16 (27)	11 (19)
Diarrhoea	21 (36)	5 (9)
Asthenia	18 (31)	2 (3)
Pain	10 (17)	3 (5)
Mucositis	11 (19)	2 (3)
Arterial hypertension	11 (19)	1 (2)
Thrombocytopenia	5 (9)	2 (3)
Anorexia	6 (10)	1 (2)
Vomiting	6 (10)	—
Nausea	6 (10)	—
Anaemia	4 (7)	1 (2)
Deep venous thrombosis	—	4 (7)
Neutropenia	1 (2)	2 (3)
Abdominal pain	3 (5)	—
Infection	3 (5)	—

Abbreviations: AE=adverse event; NCI-CTCAE=National Cancer institute Common Toxicity Criteria.
